# Supplementary material for: Relationship of mental health and burnout with empathy among medical students in Thailand: A multicenter cross-sectional study
Source: PLoS One. 2023 Jan 5;18(1):e0279564. doi: 10.1371/journal.pone.0279564 (PMC9815634; doi:10.1371/journal.pone.0279564)
Supplement: S1 Questionnaire — (DOC) [file pone.0279564.s001.doc]

**Please provide and permit information for the study.**

Mental health and burnout relate to empathy among medical students in Thailand: A multicenter cross-sectional study

Dear participants,

We are studying about mental health and burnout relate to empathy among medical students in Thailand for improve medical educational curriculum.

We would like to ask for your cooperation in answering the questionnaire without specifying name or surname into this questionnaire. We will use the obtained data to assess the overall aspect of all respondents. The decision to answer the questionnaire is voluntary, which will not have any effect to your study in medical program.

Authors

Jarurin Pitanupong, Associate Professor, M.D.

Katti Sathaporn, M.D.

Pichai Ittasakul, Associate Professor, M.D.

Nuntaporn Karawekpanyawong, M.D.

**Questionnaire**

**Part 1 Demographic information**

Please fill your information in the blank or mark 🗸 on the response form.

1. Gender ○ 1) Male ○ 2) Female
2. Age ................. years old
3. Religion ○ 1) Buddhism ○ 2) Islam ○ 3) Christianity ○ 4) Other ...............
4. Hometown ……………………………………………
5. Income ……………………….… Baht/month
6. Cumulative GPA ...................................
7. Physical illness ○ 1) No ○ 2) Yes (please identify) .........................................
8. Psychiatric illness ○ 1) No ○ 2) Yes (please identify) .........................................
9. History of alcohol use ○ 1) No ○ 2) Yes (please identify) .........................................
10. History of substance use ○ 1) No ○ 2) Yes (please identify) .........................................
11. Faculty of medicine that you have studied in

○ 1) Prince of Songkla University (Southern medical school)

○ 2) Ramathibodi Hospital, Mahidol University (Capital medical school)

○ 3) Chiang Mai University (Northern medical school)

1. Specialty preference after graduated from medical school

○ 1) General practitioner ○ 2) Pediatrician ○ 3) Ophthalmologist

○ 4) Psychiatrist ○ 5) Orthopedist ○ 6) Surgeon

○ 7) Obstetrician ○ 8) Otolaryngologist ○ 9) Internist

○ 10) Radiologist ○ 11) Anesthesiologist ○ 12) Pathologist

○ 13) Forensic pathologist ○ 14) Emergency physician ○ 15) Family physician

○ 16) Physiatrist ○ 17) Other (please identify) …….................………

1. Did you have stressor in last 1 year? ○ 1) No ○ 2) Yes (please identify in 13.1)

13.1. Please identify stressor did you have in last 1 year (you can choose more than one)

○ 1) Lesson and examination ○ 2) Atmosphere of the study

○ 3) Peer/friend problem ○ 4) Financial problem

○ 5) Family problem ○ 6) Health problem

○ 7) Other (please identify) ..............................................................................

**Part 2 Toronto Empathy Questionnaire (TEQ)**

Below is a list of statements. Please read each statement carefully and rate how frequently you feel or act in the manner of described.

Please mark  on the response form. There are no right or wrong answer or trick questions. Please answer each question as honestly as you can.

| **Statements** | 0 | 1 | 2 | 3 | 4 |
| --- | --- | --- | --- | --- | --- |
|  | **Never** | **Rarely** | **Sometimes** | **Often** | **Always** |
| 1. When someone else is feeling excited, I tend to get excited too. |  |  |  |  |  |
| 1. Other people’s misfortunes do not disturb me a great deal. |  |  |  |  |  |
| 1. It upsets me to see someone being treated disrespectfully. |  |  |  |  |  |
| 1. I remain unaffected when someone close to me is happy. |  |  |  |  |  |
| 1. I enjoy making other people feel better. |  |  |  |  |  |
| 1. I have tender, concerned feelings for people less fortunate than me. |  |  |  |  |  |
| 1. When a friend starts to talk about his\her problems, I try to steer the conversation towards something else. |  |  |  |  |  |
| 1. I can tell when others are sad even when they do not say anything. |  |  |  |  |  |
| 1. I find that I am “in tune” with other people’s moods. |  |  |  |  |  |
| 1. I do not feel sympathy for people who cause their own serious illnesses. |  |  |  |  |  |
| 1. I become irritated when someone cries. |  |  |  |  |  |
| 1. I am not really interested in how other people feel. |  |  |  |  |  |
| 1. I get a strong urge to help when I see someone who is upset. |  |  |  |  |  |
| 1. When I see someone being treated unfairly, I do not feel very much pity for them. |  |  |  |  |  |
| 1. I find it silly for people to cry out of happiness. |  |  |  |  |  |
| 1. When I see someone being taken advantage of, I feel kind of protective towards him\her. |  |  |  |  |  |

**Part 3 Thai Mental Health Indicator-15 (TMHI-15)**

Below is a list of statements. Please read each statement carefully and rate how frequently you feel or act in the manner of described in last 1 month.

Please mark  on the response form. There are no right or wrong answer or trick questions. Please answer each question as honestly as you can.

| **Statements** | **In last 1 month** | | | |
| --- | --- | --- | --- | --- |
|  | 1 | 2 | 3 | 4 |
|  | **Never** | **Rarely** | **Often** | **Always** |
| 1. I am satisfied with my life. |  |  |  |  |
| 1. I am happy. |  |  |  |  |
| 1. I am bored with my daily life. |  |  |  |  |
| 1. I am disappointed with myself. |  |  |  |  |
| 1. I feel that my life is full of sorrows. |  |  |  |  |
| 1. I can handle with intractable problems. |  |  |  |  |
| 1. I can control my emotions when trouble things happen. |  |  |  |  |
| 1. I can face with critical events in my life confidently. |  |  |  |  |
| 1. I can empathy when other people are distressed. |  |  |  |  |
| 1. I am happy when I can help other people. |  |  |  |  |
| 1. I always give other people some help when I have chance. |  |  |  |  |
| 1. I am proud of myself. |  |  |  |  |
| 1. I feel safe when I am with my family. |  |  |  |  |
| 1. I believe that my family will take care of me when I am sick. |  |  |  |  |
| 1. There is a good relationship in my family. |  |  |  |  |

**Part 4** **The Maslach Burnout Inventory (MBI)**

Below is a list of statements. Please read each statement carefully and rate how frequently you feel or act in the manner of described.

Please mark  on the response form. There are no right or wrong answer or trick questions. Please answer each question as honestly as you can.

| **Statements** | 6 | 5 | 4 | 3 | 2 | 1 | 0 |
| --- | --- | --- | --- | --- | --- | --- | --- |
|  | **Everyday** | **A few times**  **a week** | **Every week** | **A few times**  **a month** | **Monthly** | **A few times a year** | **Never** |
| 1. I feel emotionally drained from my work (study). |  |  |  |  |  |  |  |
| 1. I feel used up at the end of workday. |  |  |  |  |  |  |  |
| 1. I feel fatigued when I get up in the morning and have to face another day on the job (study). |  |  |  |  |  |  |  |
| 1. I can easily understand how my recipients (patients) feel about things. |  |  |  |  |  |  |  |
| 1. I feel I treat some recipients (patients) as if they were impersonal “objects” |  |  |  |  |  |  |  |
| 1. Working with people all day is really a strain for me. |  |  |  |  |  |  |  |
| 1. I deal very effectively with the problems of my recipients (patients). |  |  |  |  |  |  |  |
| 1. I feel burned out from my work (study). |  |  |  |  |  |  |  |
| 1. I feel I am positively influencing other people’s lives through my work (study). |  |  |  |  |  |  |  |
| 1. I have become more callous toward people since I took this job (study). |  |  |  |  |  |  |  |
| 1. I worry that this job (study) is hardening me emotionally. |  |  |  |  |  |  |  |
| 1. I feel very energetic. |  |  |  |  |  |  |  |
| 1. I feel frustrated by my job (study). |  |  |  |  |  |  |  |
| 1. I feel I am working too hard on my job (study). |  |  |  |  |  |  |  |
| 1. I do not really care what happens to some recipients (patients). |  |  |  |  |  |  |  |
| 1. Working with people directly puts too much stress on me. |  |  |  |  |  |  |  |
| 1. I can easily create a relaxed atmosphere with my recipients (patients). |  |  |  |  |  |  |  |
| 1. I feel exhilarated after working closely with my recipients (patients). |  |  |  |  |  |  |  |
| 1. I have accomplished many worthwhile things in this job (study). |  |  |  |  |  |  |  |
| 1. I feel like I am at the end of my rope. |  |  |  |  |  |  |  |
| 1. In my work (study), I deal with emotional problems very calmly. |  |  |  |  |  |  |  |
| 1. I feel recipients (patients) blame me for some of their problems. |  |  |  |  |  |  |  |

**☺ Thank you ☺**
