# Supplementary material for: Relationship of mental health and burnout with empathy among medical students in Thailand: A multicenter cross-sectional study
Source: PLoS One. 2023 Jan 5;18(1):e0279564. doi: 10.1371/journal.pone.0279564 (PMC9815634; doi:10.1371/journal.pone.0279564)
Supplement: S2 Questionnaire — (DOC) [file pone.0279564.s002.doc]

**ขอความร่วมมือในการให้ข้อมูล และขออนุญาตใช้ข้อมูลเพื่อเผยแพร่ในรูปแบบงานวิจัย**

โครงการวิจัยเรื่อง สุขภาพจิตและภาวะเหนื่อยล้าที่สัมพันธ์กับความสามารถในการเข้าใจผู้อื่นของนักศึกษาแพทย์

ในประเทศไทย: การศึกษาเชิงสำรวจภาคตัดขวางแบบพหุสถาบัน

เรียน ผู้ตอบแบบสอบถาม

เนื่องด้วยคณะผู้วิจัย มีความประสงค์ที่จะศึกษาเรื่อง สุขภาพจิตและภาวะเหนื่อยล้าที่สัมพันธ์กับความสามารถในการเข้าใจผู้อื่นของนักศึกษาแพทย์ เพื่อประโยชน์ในการพัฒนาระบบการสอนนักศึกษาแพทย์ต่อไป

จึงขอความร่วมมือจากนักศึกษา ในการตอบแบบสอบถามที่ตรงตามความเป็นจริง โดยไม่ต้องระบุชื่อ-สกุล ลงในแบบสอบถาม และสามารถส่งแบบสอบถามคืนได้ 2 ทาง ได้แก่ ส่งคืนในกล่องทึบที่จัดไว้บริเวณหน้าห้องเรียน หรือส่งคืนในกล่องทึบที่จัดไว้ที่สาขาวิชาจิตเวชศาสตร์ มหาวิทยาลัยสงขลานครินทร์ ซึ่งผู้วิจัยจะนำข้อมูลที่ได้มาประเมินเป็นภาพรวมของผู้ตอบทั้งหมด ทั้งนี้การตัดสินใจตอบแบบสอบถามให้เป็นไปด้วยความสมัครใจ ซึ่งจะไม่มีผลกระทบใดๆ ต่อการเรียนของนักศึกษาทั้งสิ้น

คณะผู้วิจัย

รศ.พญ.จารุรินทร์ ปิตานุพงศ์

อ.นพ.กัตติก์ สถาพร

รศ.นพ.พิชัย อิฏฐสกุล

อ.พญ.ณันน์ทพร การเวกปัญญาวงศ์

**คำชี้แจง**  กรุณาเขียนข้อมูลของท่านลงในช่องที่ระบุไว้ หรือใส่เครื่องหมาย ✓หน้าคำตอบที่ต้องการเลือก

**ตอนที่ 1 ข้อมูลทั่วไป**

1. เพศ ○ 1) ชาย ○ 2) หญิง
2. อายุ.................ปี
3. นับถือศาสนา ○ 1) พุทธ ○ 2) อิสลาม ○ 3) คริสต์ ○ 4) อื่นๆ ระบุ.......................
4. ภูมิลำเนาจังหวัด……………………………………………
5. รายได้ต่อเดือนของท่าน ……………………….…บาท
6. GPA สะสมในหลักสูตรแพทยศาสตร์ ...................................
7. โรคประจำตัวทางกาย ○ 1) ไม่มี ○ 2) มี (กรุณาระบุ).........................................
8. โรคประจำตัวทางจิตเวช ○ 1) ไม่มี ○ 2) มี (กรุณาระบุ).........................................
9. ประวัติการดื่มสุรา ○ 1) ไม่มี ○ 2) มี (กรุณาระบุ).........................................
10. ประวัติการใช้สารเสพติด ○ 1) ไม่มี ○ 2) มี (กรุณาระบุ).........................................
11. ท่านศึกษาอยู่สถาบันใด

○ 1) คณะแพทยศาสตร์มหาวิทยาลัยสงขลานครินทร์ ○ 2) โรงพยาบาลรามาธิบดี

○ 3) คณะแพทยศาสตร์ มหาวิทยาลัยเชียงใหม่

1. ในอนาคตท่านสนใจเรียนต่อเป็นแพทย์เฉพาะทางอยู่ในสาขาใด

○ 1) แพทย์ทั่วไป (ไม่ศึกษาต่อ) ○ 2) กุมารแพทย์ ○ 3) จักษุแพทย์

○ 4) จิตแพทย์ ○ 5) ศัลยแพทย์ออร์โธปิดิกส์ ○ 6) ศัลยแพทย์

○ 7) สูตินรีแพทย์ ○ 8) แพทย์โสต ศอ นาสิก ○ 9) อายุรแพทย์

○ 10) รังสีแพทย์ ○ 11) วิสัญญีแพทย์ ○ 12) พยาธิแพทย์

○ 13) แพทย์นิติเวช ○ 14) แพทย์เวชศาสตร์ฉุกเฉิน ○ 15) แพทย์เวชศาสตร์ครอบครัว

○ 16) แพทย์เวชศาสตร์ฟื้นฟู ○ 17) อื่นๆ ระบุ…….................………

1. ใน 1 ปีที่ผ่านมาท่านมีความเครียดหรือไม่ ○ 1) ไม่มี ○ 2) มี

14.1) ถ้ามี ท่านมีความเครียดในเรื่องใด (ตอบได้มากกว่า 1 ข้อ)

○ 1) เนื้อหาในการเรียนและการสอบ ○ 2) บรรยากาศหรือสภาพแวดล้อมในที่เรียน

○ 3) การใช้ชีวิตร่วมกับเพื่อน ○ 4) การเงิน

○ 5) ครอบครัว ○ 6) ปัญหาด้านสุขภาพของตนเอง

○ 7) อื่น ระบุ..............................................................................

**ตอนที่ 2** **แบบวัดทางจิตใจ (empathy)**

**คำชี้แจง** แบบสอบถามนี้ มีทั้งหมด 16 ข้อ โปรดเขียนเครื่องหมาย  ลงในช่อง ที่ตรงกับความรู้สึกของท่าน

| **ข้อคำถาม** | 0 | 1 | 2 | 3 | 4 |
| --- | --- | --- | --- | --- | --- |
|  | **ไม่เคย** | **แทบจะไม่** | **บางครั้ง** | **บ่อยครั้ง** | **แทบทุกครั้ง** |
| 1. เมื่อผู้อื่นรู้สึกตื่นเต้น ฉันมีแนวโน้มที่จะรู้สึกตื่นเต้นด้วย |  |  |  |  |  |
| 1. ความโชคร้ายหรือเรื่องไม่ดีที่ผู้อื่นพบเจอ ไม่ได้รบกวนจิตใจของฉันมากนัก |  |  |  |  |  |
| 1. ฉันรู้สึกไม่พอใจเมื่อเห็นผู้อื่นถูกปฏิบัติติอย่างไม่ได้รับความเคารพ |  |  |  |  |  |
| 1. ฉันรู้สึกเฉยๆ เมื่อคนใกล้ชิดของฉันมีความสุข |  |  |  |  |  |
| 1. ฉันมีความสุขที่ทำให้ผู้อื่นรู้สึกดีขึ้น |  |  |  |  |  |
| 1. ฉันรู้สึกอ่อนไหวและเป็นห่วงกังวล ต่อผู้ที่มีโชคน้อยกว่าฉัน |  |  |  |  |  |
| 1. เมื่อเพื่อนของฉันพูดถึงปัญหาของเขา ฉันพยายามที่จะขับเคลื่อนบทสนทนาไปยังทิศทางอื่นแทน |  |  |  |  |  |
| 1. ฉันสามารถบอกได้ว่าผู้อื่นกำลังโศกเศร้า ถึงแม้ว่าพวกเขาจะไม่ได้พูดออกมาก็ตาม |  |  |  |  |  |
| 1. ฉันพบว่าตัวฉันสามารถปรับอารมณ์ให้สอดคล้องกับอารมณ์ของผู้อื่นได้ |  |  |  |  |  |
| 1. ฉันไม่ได้รู้สึกเห็นใจต่อผู้ที่ทำให้ตนเองเจ็บป่วยอย่างหนัก |  |  |  |  |  |
| 1. ฉันรู้สึกหงุดหงิดเมื่อเห็นผู้อื่นร้องไห้ |  |  |  |  |  |
| 1. ฉันไม่สนใจว่าผู้อื่นรู้สึกอย่างไร |  |  |  |  |  |
| 1. ฉันมีความต้องการช่วยเหลือผู้อื่นอย่างแรงกล้า เมื่อพวกเขาเป็นทุกข์ |  |  |  |  |  |
| 1. เมื่อฉันเห็นผู้อื่นถูกปฏิบัติอย่างไม่เป็นธรรม ฉันไม่ได้รู้สึกสงสารพวกเขามากนัก |  |  |  |  |  |
| 1. ฉันพบว่ามันเป็นเรื่องงี่เง่าที่ผู้อื่นจะร้องไห้เมื่อพวกเขามีความสุข |  |  |  |  |  |
| 1. เมื่อผู้อื่นถูกลิดรอนผลประโยชน์ ฉันรู้สึกว่าต้องปกป้องพวกเขา |  |  |  |  |  |

**ตอนที่ 3** **แบบวัดความสุข**

**คำชี้แจง**  กรุณาเลือกคำตอบในช่องที่มีข้อความตรงกับตัวท่านมากที่สุด และขอความร่วมมือตอบคำถามทุกข้อคำถามต่อไปนี้จะถามถึงประสบการณ์ของท่านในช่วง 1 เดือนที่ผ่านมา ให้ท่านสำรวจตัวท่านเองและประเมินเหตุการณ์ อาการ หรือความคิดเห็นและความรู้สึกของท่าน ว่าอยู่ในระดับใดแล้วตอบลงในช่องคำตอบที่เป็นจริงกับตัวท่านมากที่สุด โดยคำตอบจะมี 4 ตัวเลือก คือ

**ไม่เลย** หมายถึง ไม่เคยมีเหตุการณ์ อาการ ความรู้สึก หรือไม่เห็นด้วยกับเรื่องนั้นๆ

**เล็กน้อย** หมายถึง เคยมีเหตุการณ์ อาการ ความรู้สึกในเรื่องนั้นๆ เพียงเล็กน้อยหรือเห็นด้วยกับเรื่องนั้นๆ

เพียงเล็กน้อย

**มาก** หมายถึง เคยมีเหตุการณ์ อาการ ความรู้สึกในเรื่องนั้นๆ มาก หรือเห็นด้วยกับเรื่องนั้นๆ มาก

**มากที่สุด** หมายถึง เคยมีเหตุการณ์ อาการ ความรู้สึกในเรื่องนั้นๆ มากที่สุด หรือเห็นด้วยกับเรื่องนั้นๆ มากที่สุด

| **ข้อคำถาม** | **1 เดือนที่ผ่านมา** | | | |
| --- | --- | --- | --- | --- |
|  | 1 | 2 | 3 | 4 |
|  | **ไม่เลย** | **เล็กน้อย** | **มาก** | **มากที่สุด** |
| 1. ท่านรู้สึกพึงพอใจในชีวิต |  |  |  |  |
| 1. ท่านรู้สึกสบายใจ |  |  |  |  |
| 1. ท่านรู้สึกเบื่อหน่ายท้อแท้กับการดำเนินชีวิตประจำวัน |  |  |  |  |
| 1. ท่านรู้สึกผิดหวังในตัวเอง |  |  |  |  |
| 1. ท่านรู้สึกว่าชีวิตของท่านมีแต่ความทุกข์ |  |  |  |  |
| 1. ท่านสามารถทำใจยอมรับได้สำหรับปัญหาที่ยากจะแก้ไข (เมื่อมีปัญหา) |  |  |  |  |
| 1. ท่านมั่นใจว่าสามารถควบคุมอารมณ์ได้เมื่อมีเหตุการณ์คับขันหรือร้ายแรงเกิดขึ้น |  |  |  |  |
| 1. ท่านมั่นใจที่จะเผชิญกับเหตุการณ์ร้ายแรงที่เกิดขึ้นในชีวิต |  |  |  |  |
| 1. ท่านรู้สึกเห็นใจเมื่อผู้อื่นมีทุกข์ |  |  |  |  |
| 1. ท่านรู้สึกเป็นสุขในการช่วยเหลือผู้อื่นที่มีปัญหา |  |  |  |  |
| 1. ท่านให้ความช่วยเหลือแก่ผู้อื่นเมื่อมีโอกาส |  |  |  |  |
| 1. ท่านรู้สึกภูมิใจในตนเอง |  |  |  |  |
| 1. ท่านรู้สึกมั่นคง ปลอดภัยเมื่ออยู่ในครอบครัว |  |  |  |  |
| 1. หากท่านป่วยหนัก ท่านเชื่อว่าครอบครัวจะดูแลท่านเป็นอย่างดี |  |  |  |  |
| 1. สมาชิกในครอบครัวมีความรักและผูกพันต่อกัน |  |  |  |  |

**ตอนที่ 4** **แบบวัดภาวะเหนื่อยล้าจากการทำงาน**

**คำชี้แจง** แบบสอบถามนี้ มีทั้งหมด 22 ข้อ โปรดเขียนเครื่องหมาย 🗸 ลงในช่อง ที่ตรงกับความรู้สึกของท่าน

ตามความหมายดังต่อไปนี้

ไม่เคยรู้สึกเช่นนั้น หมายถึง ท่านไม่เคยมีความรู้สึกเช่นนั้นเลย

อย่างมากปีละ 2-3 ครั้ง หมายถึง ท่านมีความรู้สึกเช่นนั้นอย่างมากปีละ 2-3 ครั้ง

อย่างมากเดือนละ 1 ครั้ง หมายถึง ท่านมีความรู้สึกเช่นนั้นอย่างมากเดือนละ 1 ครั้ง

เดือนละ 2-3 ครั้ง หมายถึง ท่านมีความรู้สึกเช่นนั้นเดือนละ 2-3 ครั้ง

สัปดาห์ละ 1 ครั้ง หมายถึง ท่านมีความรู้สึกเช่นนั้นสัปดาห์ละ 1 ครั้ง

สัปดาห์ละ 2-3 ครั้ง หมายถึง ท่านมีความรู้สึกเช่นนั้นสัปดาห์ละ 2-3 ครั้ง

ทุกๆ วัน หมายถึง ท่านมีความรู้สึกเช่นนั้นทุกๆ วัน

| **เกี่ยวกับความรู้สึกของท่าน** | 6 | 5 | 4 | 3 | 2 | 1 | 0 |
| --- | --- | --- | --- | --- | --- | --- | --- |
|  | **ทุกๆ วัน** | **สัปดาห์ละ**  **2-3 ครั้ง** | **สัปดาห์ละ**  **1 ครั้ง** | **เดือนละ**  **2-3 ครั้ง** | **เดือนละ**  **1 ครั้ง** | **ปีละ**  **2-3 ครั้ง** | **ไม่เคยรู้สึก**  **เช่นนั้น** |
| 1. ฉันรู้สึกจิตใจห่อเหี่ยวจากการทำงาน (การเรียน) |  |  |  |  |  |  |  |
| 1. ฉันรู้สึกหมดแรงเมื่อสิ้นสุดเวลาการทำงาน   (การเรียน) |  |  |  |  |  |  |  |
| 1. ฉันรู้สึกอ่อนเพลียเมื่อตื่นนอนตอนเช้าและรู้ว่าต้องเผชิญกับการทำงาน (การเรียน) อีกวัน |  |  |  |  |  |  |  |
| 1. ฉันสามารถเข้าถึงความรู้สึกของผู้ป่วยที่มีต่อสิ่งต่างๆ ได้ง่าย |  |  |  |  |  |  |  |
| 1. ฉันรู้สึกว่าได้ปฏิบัติต่อผู้ป่วยบางคนเสมือนเขาเป็นสิ่งที่ไร้ชีวิตจิตใจ |  |  |  |  |  |  |  |
| 1. การทำงาน (การเรียน) เกี่ยวกับการบริการผู้คนตลอดวันเป็นสิ่งที่ตึงเครียดสำหรับฉัน |  |  |  |  |  |  |  |
| 1. ฉันสามารถแก้ปัญหาของผู้ป่วยได้อย่างมีประสิทธิภาพ |  |  |  |  |  |  |  |
| 1. ฉันรู้สึกเหนื่อยหน่ายในภาระงาน (เรียน) |  |  |  |  |  |  |  |
| 1. ฉันรู้สึกแน่ใจว่างาน (การเรียน) ของฉันมีอิทธิพลต่อชีวิตของผู้อื่น |  |  |  |  |  |  |  |
| 1. ฉันเปลี่ยนเป็นคนหยาบกระด้างและไร้เมตตาต่อผู้อื่นมากขึ้นตั้งแต่ทำงาน (เรียน) นี้ |  |  |  |  |  |  |  |
| 1. ฉันวิตกกังวลว่า งาน (การเรียน) กำลังทำให้จิตใจของฉันแข็งกระด้างขึ้น |  |  |  |  |  |  |  |
| 1. ฉันรู้สึกเปี่ยมไปด้วยพลัง |  |  |  |  |  |  |  |
| 1. ฉันรู้สึกคับข้องใจในการทำงาน (การเรียน) |  |  |  |  |  |  |  |
| 1. ฉันรู้สึกว่ากำลังทำงาน (เรียน) ที่หนักมากเกินไป |  |  |  |  |  |  |  |
| 1. ฉันรู้สึกว่าตัวเองไม่ใส่ใจว่าอะไรจะเกิดขึ้นกับผู้ป่วยบางคน |  |  |  |  |  |  |  |
| 1. การดูแลผู้ป่วยโดยตรงทำให้ฉันรู้สึกเครียดมากเกินไป |  |  |  |  |  |  |  |
| 1. ฉันสามารถสร้างบรรยากาศที่ผ่อนคลายในการดูแลผู้ป่วยได้ง่าย |  |  |  |  |  |  |  |
| 1. ฉันรู้สึกเป็นสุขใจภายหลังการดูแลผู้ป่วย |  |  |  |  |  |  |  |
| 1. ฉันได้สร้างสรรค์สิ่งที่มีคุณค่ามากมายในการปฏิบัติงาน |  |  |  |  |  |  |  |
| 1. ฉันรู้สึกสิ้นหวัง |  |  |  |  |  |  |  |
| 1. ในการทำงานฉันสามารถเผชิญปัญหาทางอารมณ์ได้อย่างสงบ |  |  |  |  |  |  |  |
| 1. ฉันรู้สึกว่าได้รับการตำหนิจากผู้ป่วย ในปัญหาบางอย่างที่เกิดขึ้น |  |  |  |  |  |  |  |

**☺ขอบคุณที่ให้ความร่วมมือในการตอบแบบสอบถาม**☺
