# Supplementary material for: Relationship of mental health and burnout with empathy among medical students in Thailand: A multicenter cross-sectional study
Source: PLoS One. 2023 Jan 5;18(1):e0279564. doi: 10.1371/journal.pone.0279564 (PMC9815634; doi:10.1371/journal.pone.0279564)
Supplement: Attachment — Submitted filename: Response to reviewers.docx [file pone.0279564.s004.docx]

Dear, editor and reviver team

Thank you for your kindly review, comments, and suggestion.

Author's Responses to Questions of reviewer

**Reviewer #1: Dear Authors,**

**Q:** I am delighted to see a study on mental health and burnout from Thailand. Appropriate, well-validated (within a North American context) questionnaires were utilized to assess whether there is an association with empathy, broadly speaking, burnout, mental health, as well as what I perceive to be 'control' variables, such as substance use within your demographics questionnaire. **In North American studies, what we do find is generally a decrease in empathy (per scale used for that study), and generally a negative relationship between burnout and empathy.** However, given that your study focuses on Thai **medical students**, these constructs **may not hold the same sort of validity**, or may need to **be interpreted differently**. Indications for me was 1) high personal achievement despite burnout, which is fairly surprising given the North American literature, and 2) the conflation of all three aspects of empathy to be equally important, when they may be best served as broken apart into cognitive v emotional empathy. There is a great deal of **sociological/philosophical literature and debate on the utilization of emotional empathy (see Paul Bloom's Against Empathy)** and whether it is even needed within the clinical encounter.

A: **Thanks for your kindly and useful recommendation.**

**We remove** **the sentence or wording that may be too speculative or not relate to the evidence of this research as the following**

This variance might be due to differences in study instruments, ~~population ethnicity, and culture~~.

**We add discussion related to Paul Bloom's Against Empathy [reference 42] as following**

However, empathy and concern other’s mind may be psychologically distinct and empathy may be limited by our moral lives [42].

**Q:** I recommend to the authors to do their analysis but breaking both burnout and empathy constructs apart into their three dimensions to better understand the reasons for the differences seen. Merely stating that they are cultural differences without pulling in evidence suggestive of such (in Thai, non-medical student samples) is far too speculative.

**A: We try to do more analysis by breaking both empathy constructs apart and burnout into three dimensions as below.**

However, three dimensions of burnout (EE, DP, PA) were evaluated to determine if they had an impact on any subgroups of empathy. Data was analyzed using a linear regression for each of only two dimensions of burnout (EE and DP) because of the majority of participants (97%) reported high PA. It was discovered that participants with higher levels of DP had statistically lower scores of demonstrating appropriate sensitivity, altruism, and behaviors engaging higher-order empathic responding (p<0.001, p<0.003, and p<0.014, respectively).

**Reviewer #2:** Thank you for submitting this manuscript reporting results of your study of three Thai medical school students' self-reported mental health, burnout and empathy. The following comments are meant to help the authors refine their work. I feel these edits will be easy for the authors to make.

**Thanks for your kindness review**

**Abstract**

**Q:** At the conclusion of your results and in the conclusions, you suggest that the statistical relationship between the mental health scale with empathy is causal. The analysis indicates there is a positive association between the two measures, **but it does not mean that better mental health improves empathy levels.** The wording needs to be revised to reflect that.

**A: We correct the conclusion according your suggestion as**

Most medical students had below-average empathy levels, and two-thirds of them had high emotional exhaustion levels, yet most of them reported having a high level of personal accomplishment and good mental health. ~~Better mental health resulted a protective factor that improves the level of empathy~~ There was an association between mental health and the level of empathy. Higher levels of depersonalization related to lower scores of demonstrating sensitivity, altruism, and behaviors responding.

**Introduction**

**Q: Line 96-98**: I am not entirely convinced this sentence belongs here. It read as something that should be part of the conclusions of your manuscript and not the background. It may be worth discussing amongst yourselves or the editor to decide.

**A: We remove all these sentence as suggest.**

~~The findings of this study provide useful information for efforts to enhance empathy, promote well-being, and reduce distress among medical students as well as establish educational programs in the medical curriculum geared at boosting medical professionalism.~~

**Materials and Methods**

**Q:** With regard to the administration of the questionnaire, I was curious if there was a plan of **who would get the paper questionnaire and who would get the electronic version.** The reason I ask is that what if you administer it to a class on paper and the student forgets they did that and completes the electronic version as well. I know it is unlikely, but it is possible. A better explanation of **how these two administrations were done and why is needed**.

**A: We add the more explanation of these issue as**

“The data were collected in the given classroom following relevant guidelines by using a paper-based or online process. One participant had the right to choose only one way to answer the questionnaire. Before entering the study, all participants were asked whether responded on paper or online questionnaire to prevent double response of completing this study.”

**Q:** For the section on questionnaires, TMHI-15 and MBI need to have a sentence similar to the TEQ that defines item response options and scores. Along those same lines, it would be helpful to have how many questions on MBI and TEQ are included in their subscores. You indicated scores for MBI, but that does **not tell us how many questions** nor the scale.

I also had a question about MBI. Is there an expected correlation between the three subscales? It seems that emotional exhaustion and depersonalization would have a positive correlation and the inverse with personal accomplishment. It may be worth noting what should be expected and if your study found similar results.

**A: We add the more explanation of the range of each sub-scores in TMHI, MBI Tool as**

2) Thai Mental Health Indicator-15 (TMHI-15) consisted of 15 questions. The score of each question ranged from 1 to 4; 1 (never); 2 (rarely); 3 (sometimes); and 4 (always),

3) Thai version of the Maslach Burnout Inventory (MBI) questionnaire [29,30] consisted of 22 items divided into three dimensions: emotional exhaustion (feelings of being emotionally overextended and exhausted by one’s work), depersonalization (unsympathetic and impersonal responses toward the recipients of one’s care or service), and personal accomplishment (feelings of competence and achievement in one’s work with people) [30]. Emotional exhaustion (EE) subscale ranged from 0 (never) to 6 (every day), Cronbach’s alpha coefficient=0.9. Depersonalization (DP) subscale ranged from 0 (never) to 6 (every day), Cronbach’s alpha coefficient=0.7. Personal accomplishment (PA) subscale ranged from 0 (every day) to 6 (never), Cronbach’s alpha coefficient=0.7.

**Results**

**Q:** section read nicely.

**A:** **We feel good, thank you so much ^__^**

**Discussion**

**Q:** Some additional limitations that should be addressed include **potential bias due to greater number of female respondents** and **large number of Buddhist respondents**. Also, have you considered **non-response bias** for those students not completing the survey?

**A: We add the limitation according to suggestion as**

Additionally, there might have potential bias due to greater number of female respondents and large number of Buddhist respondents.

**Q:** Also, have you considered **non-response bias** for those students not completing the survey?

**A: We considered non-response bias for those students who not completing the survey, then we add more explanation in difference of demographic characteristic as**

Additionally, there was no statistically significant difference in demographic data (gender, religion) between participants and non-participants.

**Conclusion**

**Q:** Line 301 should be modified per my comment about the abstract.

**A:** **We correct the conclusion according the suggestion as**

~~Better mental health was found to be a significant protective factor for improving the level of empathy.~~ There was the association between mental health and the level of empathy. Higher levels of depersonalization related to lower scores of demonstrating sensitivity, altruism, and behaviors engaging empathic responding.

**Finally**

We have sent our “Revised manuscript” for re-checked and re-proofreading the English from the Office of International Affairs of the Faculty of Medicine.

Sincerely thanks for every nice, kind comments and suggestion, they are very useful to improve our manuscript.

Best regards
