# Supplementary material for: Relationship of mental health and burnout with empathy among medical students in Thailand: A multicenter cross-sectional study
Source: PLoS One. 2023 Jan 5;18(1):e0279564. doi: 10.1371/journal.pone.0279564 (PMC9815634; doi:10.1371/journal.pone.0279564)
Supplement: Attachment — Submitted filename: response to reviewer V3.docx [file pone.0279564.s005.docx]

Response to review Comments

Title: Relationship of mental health and burnout with empathy among medical students in Thailand: A multicenter cross-sectional study.

Reviewer #3: Major revision is needed

C: One of the objectives is to determine the association between burnout and empathy: So, it is not true to adjust for burnout in logistic regression. Burnout must be included in the model beside mental health.

It is not true to make two models (one for personal and one for depersonalization): you should make one logistic regression model that includes mental health, depersonalization and personal exhaustion. I mean the relation between burnout and empathy should be clearly reported in the logistic regression.

In your models (table 3 and 4), I noticed that you report one p value; it should be two p values: one for each category as compared with the reference group. Do this for the new model.

The sequence of table is not correct: begin with all the descriptive analysis then move to bivariate and then to multivariate: so, table one should include only descriptive statistics. Put the association just before the last table which is the multiple logistic regression.

A: We have modified the manuscript and tables as suggested (as table 1 show only descriptive data, and table 2,3 show bivariate and then multivariate, respectively)

C: If there are new findings, discuss them and include them in the conclusion.

A: The finding is not different from the original or previous results.

C: Have you checked for multicollinearity in multiple logistic regression?

A: Yes, we check it. Thanks.

C: If your conclusion does not reflect the findings. Please rewrite it.

A: The finding is the same as the prior results, then the conclusion is the same.

C: After the conclusion, write your recommendation according to your findings.

A: The finding is the same as the prior results. However, we concern about your valuable suggestion, then we add our recommendation according to our findings in conclusion part “Therefore, medical educators should pay close attention to promoting good mental health among medical students.”

Finally, we are grateful for your good wishes and valuable suggestion. This is helpful so much and encourages us to make this manuscript to get better. We wish to have more best wishes from you again. Thanks.
